# Traumatic inferior lumbar hernia

**DOI:** 10.1093/jscr/rjac240

**Published:** 2022-05-27

**Authors:** Niyaz Baig, Hassan Elberm, Patrick Warren

**Affiliations:** University Hospital Southampton, Acute Surgical Unit, General Surgery, Tremona Road, Southampton, Hampshire, SO 16 6YD, UK; University Hospital Southampton, Acute Surgical Unit, General Surgery, Tremona Road, Southampton, Hampshire, SO 16 6YD, UK; University Hospital Southampton, Acute Surgical Unit, General Surgery, Tremona Road, Southampton, Hampshire, SO 16 6YD, UK

## Abstract

Traumatic lumbar hernias are rare, with little surgical literature describing their management. In this we report about a 41-year-old patient admitted to University Hospital Southampton following a high velocity road traffic collision, of estimated speed 140 mph. He suffered multiple bony injuries, and a traumatic right inferior lumbar hernia containing the ascending colon. The hernia was surgically repaired extra-peritoneally with mesh and abdominal wall muscles approximated and sutured.

## INTRODUCTION

The two lumbar triangles are situated between the bottom of the 12th (last) rib and the iliac crest. These are the sites of the two respective types of lumbar hernias: one protrudes through the superior lumbar triangle and the other protrudes through the inferior lumbar triangle [1]. The inferior lumbar triangle is bound by the free border of the latissimus dorsi-posteromedially, the external oblique-anterolaterally and the iliac crest-inferiorly. Superior lumbar triangle is bounded by the 12th rib superiorly, quadrates lumborum medially, laterally by the internal oblique muscle [2]. Herniation through this triangle is rare and is known as Petit’s hernia or inferior lumbar hernia. Roughly 25% of lumbar hernias are secondary (e.g. traumatic or post-infection), 55% are spontaneous (primary acquired) and 20% arise congenitally [1–5].

## CASE REPORT

**Figure 1:**
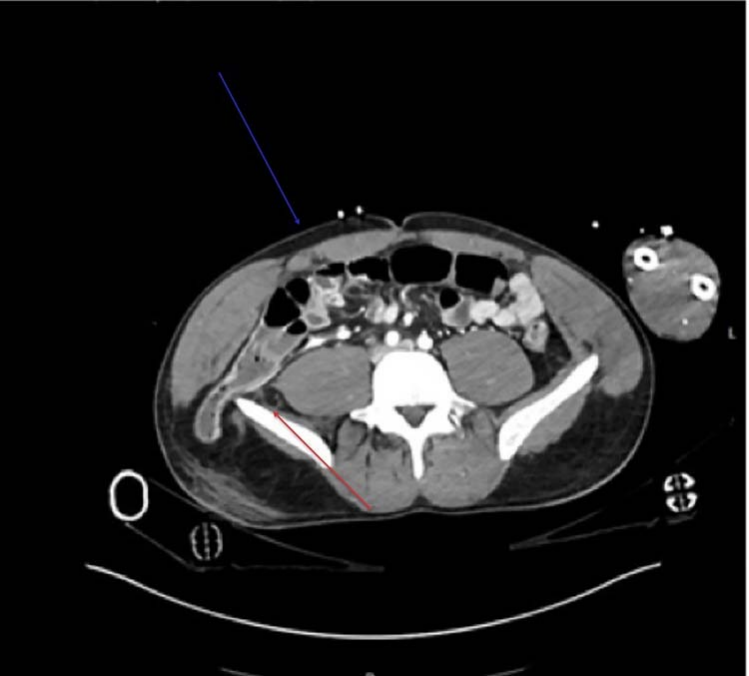
Axial view of the CT demonstrates the tear in the rectus muscle (blue arrow). Inferior lumbar hernia can be clearly demonstrated, above the iliac crest (red arrow).

**Figure 2:**
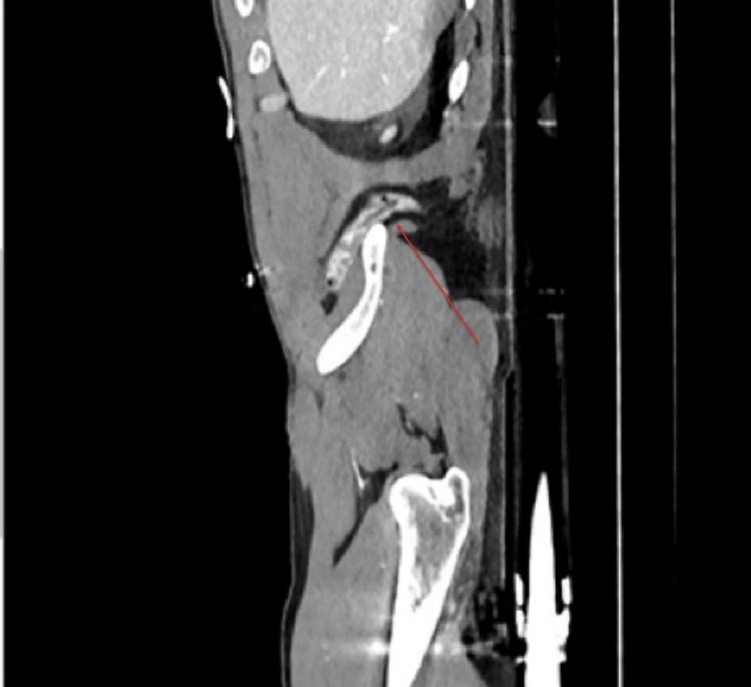
Sagittal view demonstrates the inferior lumbar hernia (red arrow).

**Figure 3:**
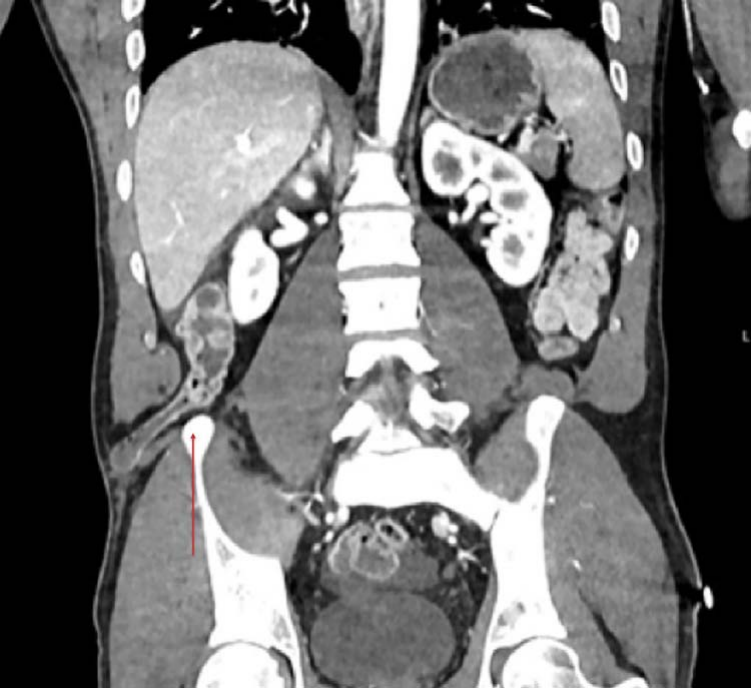
Coronal view showcases the inferior lumbar hernia above the iliac crest (red arrow).

Traumatic lumbar hernias are quite uncommon, as such most surgeons are unfamiliar with them. This was a 41-year-old male Caucasian who was involved in a head on vehicular collision of estimated velocity of 140 mph. Computed tomography (CT) trauma scan confirmed a lumbar hernia through the inferior triangle of petit containing the ascending colon (Figs 1–3). Multiple bony fractures were also noted on the imaging. In discussion regarding the management of the hernia, it was felt that urgent repair was needed to avoid bowel ischaemia or perforation, rather than delayed repair. To repair the defect a laparoscopic approach was initially trialled, (transabdominal repair). Peritoneum was incised around the hernial defect to access the extra-peritoneal space. The hernial sac was dissected; bowels showed no macroscopic signs of ischaemia or injury in the sac. However there was major avulsion of muscles from the iliac crest (>15-cm defect; Fig 1), which was closed posteriorly. Anteriorly it was difficult to close the defect as it was difficult to suture the muscles on to the periosteum of the iliac crest. A decision was made to convert to open surgery as to better access the muscles for suturing. Ventralight mesh was placed extra-peritoneally for further enforcement. The right inferior epigastric vessels were also notably torn, which were tied. Torn right rectus muscles were also approximated and sutured. Peritoneum was closed. This repair was performed extra-peritoneally.

## DISCUSSION

Due to the rarity of traumatic lumbar hernias, the appropriate time frame for repair is currently not well established in surgical literature.

Lumbar hernias are often asymptomatic. Diagnosis is usually established by the presence of a reducible soft-tissue mass above the iliac crest, which enlarges with the Valsalva maneuver or coughing [5].

Lumbar hernias may contain peritoneum and intra-peritoneal organs, or may be entirely extra-peritoneal.

In the absence of an agreed approach to manage these hernias, in light of contained bowels in this instance, urgent repair was warranted rather than a delayed approach.

## CONSENT

Consent was given by the patient, all details anonymised and patient identifiers omitted. The patient agreed and signed a consent form allowing the use of images and description of the event and procedures to be published. Patients’ personal health identifiers are omitted.

## CONFLICT OF INTEREST STATEMENT

None declared.

## FUNDING

None.
